# Small Extracellular Vesicles from Human Fetal Dermal Cells and Their MicroRNA Cargo: KEGG Signaling Pathways Associated with Angiogenesis and Wound Healing

**DOI:** 10.1155/2020/8889379

**Published:** 2020-08-13

**Authors:** Cinzia Maria Chinnici, Giandomenico Amico, Alessia Gallo, Gioacchin Iannolo, Nicola Cuscino, Serena Vella, Claudia Carcione, David Nascari, Pier Giulio Conaldi

**Affiliations:** ^1^Fondazione Ri.MED, Palermo, Italy; ^2^Department of Research, IRCCS-ISMETT, Palermo, Italy; ^3^Innovation and Development Department, Anemocyte Srl, Gerenzano, Italy; ^4^McGowan Institute for Regenerative Medicine, University of Pittsburgh, Pittsburgh PA, USA

## Abstract

The use of cell secreted factors in clinical settings could be an alternative to conventional cell therapy, with the advantage of limiting concerns generally associated with traditional cell transplantation, such as tumorigenicity, immunoreactivity, and carrying of infections. Based on our published data, we predict a potential role for extracellular vesicles (EVs) in contributing to the proangiogenic activity of human fetal dermal cell secretome. Depletion of nanosized EVs from secretome significantly impaired its ability to induce formation of mesh-like structures *in vitro*. The isolated EVs were characterized for size and concentration by nanoparticle tracking analysis, and for protein markers (Rab5^+^, Alix^+^, CD63^+^, and calnexin^−^). The microRNA profile of EVs revealed 87 microRNAs significantly upregulated (≥15-fold increase) in fetal compared to adult dermal cell-derived EVs. Interestingly, these upregulated microRNAs included microRNAs with a validated role in angiogenesis according to literature. Moreover, the DIANA-TarBase v7.0 analysis confirmed enrichment in the KEGG signaling pathways associated with angiogenesis and wound healing, with the identification of putative target genes including thrombospondin 1. To validate the *in silico* data, EVs were also characterized for total protein contents. When tested in *in vitro* angiogenesis, fetal dermal cell-derived EVs were more effective than their adult counterpart in inducing formation of complete mesh-like structures. Furthermore, treatment of fibroblasts with fetal dermal-derived EVs determined a 4-fold increase of thrombospondin 1 protein amounts compared with the untreated fibroblasts. Finally, visualization of CSFE-labeled EVs in the cytosol of target cells suggested a successful uptake of these particles at 4-8 hours of incubation. We conclude that EVs are important contributors of the proangiogenic effect of fetal dermal cell secretome. Hence, EVs could also serve as vehicle for a successful delivery of microRNAs or other molecules of therapeutic interest to target cells.

## 1. Introduction

The list of clinical conditions related to insufficient angiogenesis is wide, ranging from cardiovascular diseases to impaired wound healing [1]. Therefore, there is a great interest in developing clinical strategies ensuring vasculature formation, such as delivery of different cell populations, or administration of proangiogenic growth factors. Among the cell-based therapies, the use of mesenchymal stromal cells (MSCs), which are closely related to pericytes and produce diverse proangiogenic factors, is a promising approach with the potential to stimulate vasculature tissue development [2].

MSC-based therapy applied to regenerative medicine counts hundreds of registered clinical trials with excellent records of safety and efficacy (http://www.clinicaltrials.gov and http://www.clinicaltrialsregister.eu). Despite earlier works ascribing the therapeutic effects of MSCs to their ability to engraft and differentiate to form new permanent tissues, the current consensus view is that MSCs are short-lived after delivery and exert therapeutic benefits through secretion of bioactive factors [3]. In support of MSC paracrine activity, animal studies have shown that administration of MSC-derived soluble factors recapitulates the effects of cell-based therapy [4]. Consequently, the attention has been brought to the vast array of molecules produced by MSCs [5, 6]. The mixture of growth factors, cytokines, chemokines, and extracellular vesicles (EVs) released by cells is known as secretome and can be collected as cell culture conditioned medium (CM).

Human fetal skin cell therapy has been used to replace older skin cell therapy to treat patients with skin ulcers [7, 8], as well as burns [9, 10], thus resulting in a safe and more efficacious procedure. At that time, the authors suggested the differential gene expression profiling observed in fetal versus adult skin cells as responsible for the efficacy of fetal skin cell therapy [8]. Moreover, since no trace of fetal skin cells was found in recipient biopsies, a paracrine mechanism of healing was suggested [10]. Although an extensive characterization of cell phenotype and secreted factors was missing, those studies open up new research perspectives on the use of cell secretome for regenerative medicine applications.

In a previous study [11], we isolated human fetal dermal cells, which we named multipotent fetal dermal cells based on *in vitro* characteristics, such as the MSC-like immunophenotype, the multilineage differentiation potential, and the low immunogenicity. We also confirmed the practical advantages of culturing fetal skin cells in comparison to adult skin cells described by others [7, 9, 10], including faster isolation technique and higher proliferative capacity of fetal cells. More recently [12], we used liquid chromatography and tandem mass spectrometry (LC-MS/MS) to show a set of proteins related to angiogenesis and wound healing, which were significantly upregulated in fetal dermal cell secretome compared to adult dermal cell secretome. Proteome finding was corroborated by the remarkable *in vitro* proangiogenic capacity of fetal dermal cell secretome compared to its adult counterpart.

In the present study, we investigated whether the presence of EVs could contribute to the biological functions of fetal dermal cell secretome. EVs are released by several cell types and are essential for cell-to-cell communication [13]. These particles are internalized by target cells [14] and once in the cytosol, discharge their material such as proteins, mRNAs, and microRNAs (miRNAs). Administration of MSC-derived EVs has been shown to have beneficial effects in various animal models of organ injury by regulating angiogenesis, cell proliferation, cell migration, and collagen synthesis [15, 16]. Nevertheless, EV-based therapy for skin repair consists only of one registered clinical trial aims at studying the effects of plasma-derived exosomes on cutaneous wound healing (http://www.ClinicalTrials.gov, NCT02565264).

Herein, EVs were isolated from secretome of fetal and adult dermal cells and characterized for size and concentration by nanoparticle tracking analysis (NTA). Then, we tested the capacity of EV-depleted secretome in inducing *in vitro* angiogenesis and migration of target cells such as human umbilical vein endothelial cells (HUVECs) and fibroblasts. The miRNA expression profile of fetal dermal cell- vs. adult dermal cell-derived EVs was also analyzed, and a bioinformatics approach was used to identify the Kyoto Encyclopedia of Genes and Genomes (KEGG) signaling pathways most likely affected by these miRNAs. The predicted angiogenic/wound healing-related effects of fetal dermal cell-derived EVs were further validated in *in vitro* cell-based assays of angiogenesis and cell migration by preconditioning of target cells with different concentrations of EV preparations. Finally, we investigated whether carboxyfluorescein succinimidyl ester- (CSFE-) labeled EVs from fetal dermal cells might be taken up by target cells.

## 2. Materials and Methods

### 2.1. Cell Procurement

Fetal skin biopsies were taken from 20- to 22-gestational-week human fetuses obtained from therapeutic abortions, according to a protocol approved by ISMETT's Institutional Research Review Board (IRRB/00/2015) and Ethics Committee. Signed informed consent form was obtained from each donor. Fetal dermal cells were isolated and characterized as previously described [11]. Adult skin biopsies (45–55-year-old donors) were provided by Istituto Humanitas (Rozzano, Milan). Fetal and adult dermal cells were isolated under the same conditions. Normal dermal fibroblasts and HUVECs were purchased from Thermo Fisher Scientific (Waltham, MA, USA) and used as target cells in *in vitro* cell-based assays.

### 2.2. Cell Cultures, CM Collection, Isolation, and Storage of EVs

Fetal dermal cells and adult dermal cells, previously cryopreserved in Dulbecco's modified Eagle's medium (DMEM) (Sigma-Aldrich, St. Louis, MO, USA) supplemented with 10% dimethyl sulfoxide (DMSO) (CryoSure, WAK-Chemie Medical GmbH, Steinbach, Germany) and 30% fetal bovine serum (FBS), were grown in 75 cm^2^ tissue flasks (SARSTEDT, Numbrecht, Germany) as previously described [11]. For CM collection, serum-free alpha-minimum essential medium (MEM) (Gibco, Thermo Fisher Scientific) was added to 80% confluent cells and collected 24 hours later. EVs were isolated by differential ultracentrifugation of CM according to a protocol [17] and as previously described [18]. In brief, CM was centrifuged at 1800 × *g* for 10 minutes to remove cell debris, centrifuged again at 17000 × *g* for 15 minutes, and then at 160000 × *g* for 1 hour in the ultracentrifuge (Optima MAX-XP, Beckman Coulter Inc., Irving, TX, USA). All centrifugation steps were done at 4°C. The pellet was resuspended in phosphate-buffered saline (PBS) without Ca^2+/^Mg^2+^ (Sigma-Aldrich) or subjected to total protein extraction or total RNA extraction.

### 2.3. Characterization of EVs by Nanoparticle Tracking Analysis

Pellet particles resuspended in PBS (three fetal and three adult samples) were analyzed for size and concentration by nanoparticle tracking analysis (NTA) [19] using the NanoSight (NS300, Malvern Instruments, Westborough, MA, USA). Briefly, samples were diluted in PBS, 300 *μ*l of sample was loaded into the chamber, and five videos for each sample were recorded. Data analysis was performed with the NTA software and data were presented as the mean ± standard deviation (SD) of the five videos.

### 2.4. Extraction of Total RNA from EVs, Reverse Transcription (RT), qPCR, and TaqMan Low-Density Arrays (TLDA) for miRNA Profiling

Total RNA was extracted from EVs of fetal and adult dermal cells using the miRNeasy Mini Kit (Qiagen, Hilden, Germany), according to manufacturer's instructions. The purity of isolated RNA was determined by OD_260/280_ using a NanoDrop (ND-1000, Thermo Fisher Scientific). Reverse transcription (RT) and preamplification were done using the High-Capacity cDNA Reverse Transcription Kit (Applied Biosystems, Thermo Fisher Scientific) according to manufacturer's instructions. The kit includes the Megaplex PreAmp Primers Human Pool A v2.1 and the Megaplex PreAmp Primers Human Pool B v3.0 (both primers from Applied Biosystems, Thermo Fisher Scientific). miRNA profiling of three fetal vs. three adult EV preparations was done with TaqMan Array Human MicroRNA A+B Cards (Life Technologies, Carlsbad, CA, USA), which analyzes 754 human miRNAs. PCR was done with the Applied Biosystems 7900 HT Real-Time PCR system. The expression level of each miRNA was determined by equation 2^-*ΔΔ*CT^. Student's *t*-test was used to calculate the *p* value, and the threshold was set at ≤0.05. Data were considered significant at a fold change > 15. Furthermore, significantly upregulated miRNAs in fetal vs. adult samples were screened out with the online prediction software program DIANA-miRPath v.3 [20]. We selected DIANA-TarBase v7.0 for analysis and set the *p* value ≤ 0.05 to analyze miRNAs and their target genes. KEGG enrichment analysis was used to identify signaling pathways most enriched by our miRNAs. In order to identify single genes targeted by multiple miRNAs, KEGG analysis was performed with the “genes intersection” option.

### 2.5. Quantification of EV Total Protein Contents

Total protein extraction from EVs was done with radioimmunoprecipitation assay (RIPA) buffer (Thermo Fisher Scientific) supplemented with halt protease and phosphatase inhibitors cocktail (Thermo Fisher Scientific). Tissue extracts were centrifuged at 12000 × *g* for 15 minutes at 4°C. Total protein contents were quantified with the bicinchoninic acid (BCA) assay (Thermo Fisher Scientific) by using the Tecan Spark 10 M microplate reader (BioExpress, VWR, Radnor, PA, USA). Both freshly isolated and frozen EV preparations were used in functional assays.

### 2.6. Western Blot Analysis

For biochemical characterization of EVs, 30 *μ*g/lane of total protein extracts was separated by sodium dodecyl sulfate- (SDS-) polyacrylamide gel electrophoresis and transferred to nitrocellulose membranes (Bio-Rad Laboratories, Richmond, CA, USA). The membranes were blocked with 5% nonfat milk in T-TBS (50 mmol/l Tris pH 7.5, 0.9% NaCl, and 0.1% Tween-20) (all from Sigma-Aldrich) overnight at 4°C and incubated 1 hour at room temperature with the following primary antibodies: mouse monoclonal antibody raised against recombinant human Rab5 (F-9, sc-373725, Santa Cruz Biotechnology, Santa Cruz, CA, USA; 1 : 500 dilution), mouse monoclonal antibody against Alix (3A9, 2171, Cell Signaling Technology, Danvers, MA, USA; 1 : 1000 dilution), and mouse monoclonal antibody against CD63 (MX-49.129.5, sc-5275, Santa Cruz Biotechnology; 1 : 200 dilution). As a negative marker of EVs, a rabbit monoclonal antibody against calnexin (C5C9, 2679, Cell Signaling; 1 : 1000 dilution) was used. For this latter, total protein extracts from cells were also included in the analysis.

For validation of the *in silico* analysis, fibroblasts were treated with 10 *μ*g/ml of fetal-derived EVs for 24, 48, and 72 hours. 30 *μ*g/lane of fibroblast total protein extracts was separated by SDS polyacrylamide gel electrophoresis and transferred to nitrocellulose membranes (Bio-Rad Laboratories). The membranes were incubated 1 hour at room temperature with a rabbit monoclonal antibody against recombinant human thrombospondin 1 (THBS1) (ab267388, Abcam, Cambridge, UK; 1 : 1000 dilution). Beta-actin was used as an internal loading control (sc-81178, Santa Cruz Biotechnology; 1 : 1000 dilution). After three washings with T-TBS, the membranes were incubated for 1 hour at room temperature with horseradish peroxidase-conjugated secondary antibodies (Santa Cruz Biotechnology; 1 : 10000 dilution). After washing, the signal was detected with an enhanced chemiluminescence reagent (ECL; Amersham, Arlington Heights, IL, USA). Densitometric analysis of Western blot analysis was done with the Image Lab software, version 6.0.1 (Bio-Rad Laboratories).

### 2.7. EV/CM-Induced *In Vitro* Angiogenesis

Serum-starved HUVECs were resuspended in serum-free culture medium supplemented with different concentrations of EVs (100, 50, 10, or 5 *μ*g/ml) from fetal and adult dermal cells. Approximately 10000 cells/well were plated in triplicate onto Matrigel from the *in vitro* angiogenesis assay kit (Millipore, Billerica, MA, USA) in flat bottom 96-well plastic plates (Costar Corning Inc., Costar, NY, USA). HUVECs plated in fetal dermal cell CM or in serum-free culture medium were used as positive and negative control, respectively. Plates were incubated at 37°C in a humidified atmosphere with 5% CO_2_. Formation of mesh-like structures was monitored for 24 hours with an inverted microscope (Olympus CKX41, Tokyo, Japan) coupled with a camera (Olympus U-TV0.5XC-3) for image acquisition. Numerical values (score from 0 to 5) were assigned to each pattern according to manufacturer's specifications (Millipore) and as previously described [12]. Formation of mesh-like structures was quantified by calculating the number of junctions, nodes, total mesh area, and total segments length, with ImageJ software of the Angiogenesis Analyzer plugin (https://imagej.nih.gov/ij/).

### 2.8. *In Vitro* EV/CM-Induced Cell Migration

Cell migration was monitored by using the Cellular Invasion/Migration (CIM) Plate 16 with the XCELLigence Real-Time Cell Analyzer (RTCA) dual purpose (DP) instrument (Acea Biosciences Inc., San Diego, CA, USA), which detects the real-time migration of cells [21]. Briefly, serum-starved fibroblasts were resuspended in culture medium supplemented with 100, 50, 10, or 5 *μ*g/ml EVs from fetal and adult dermal cells or in culture medium without EVs, and added to the upper chamber (30000 cells/chamber) of the CIM plate. Culture medium 0.5% FBS was used as chemoattractant and loaded to the lower chamber. The CIM plates were assembled into the RTCA-DP instrument and placed in the incubator at 37°C in a humidified atmosphere with 5% CO_2_. Cell migration was recorded every 15 minutes for 7 hours. Each time point was calculated from duplicate values, and cell migration was expressed as cell index (CI) at a 7-hour time point. Analysis was performed with the RTCA Software 1.2 of the xCELLigence system.

### 2.9. Quantitative Analysis of EV-Depleted CM by Luminex Technology

EV-depleted CM from fetal dermal cells was subjected to quantitative evaluation by Luminex xMAP technology (Luminex 200; Luminex Corp., Austin, TX, USA), enabling the simultaneous detection of analytes. The analyzed soluble factors included human growth factors such as VEGF-A and HGF, and chemokines with a documented role in angiogenesis and wound healing, such SDF-1 alpha, MCP-1, IL-8, and GRO-alpha. These factors were included in a customized panel (ProcartaPlex, Thermo Fisher Scientific). Briefly, undiluted or 1 : 10 diluted CM was loaded into the multiplex and processed according to manufacturer's instructions. Concentration of soluble factors was calculated by using software provided by the manufacturer, and the results normalized to the total number of attached cells. The concentration of soluble factors was expressed as pg/ml/1.5 × 10^6^ cells/24 hours. Data from EV-depleted CM were compared with those from whole CM.

### 2.10. EV Labeling and Cellular Uptake Assay

EVs from fetal dermal cells were labeled with CSFE (Thermo Fisher Scientific) according to manufacturer's instructions with minor modifications. Briefly, 1 : 1000 diluted CSFE was added to 10 *μ*g of EV preparation and incubated at 37°C for 15 minutes. 1 ml of 1% bovine serum albumin (BSA) (Sigma-Aldrich) was added to stop the labeling, and the mixture was ultracentrifuged at 100000 × *g* for 70 minutes at 4°C. The supernatant was discharged, the pellet resuspended in serum-free DMEM, and EV labeling was verified by flow cytometry with a FACS Canto II (Becton Dickinson, BD, Franklin Lake, NJ, USA) in a log range by using 50 nm diameter reference beads as size standard (MicroBeads, Miltenyi, Bergisch Gladbach, Germany), as previously described [22]. Fibroblasts and HUVECs previously grown to 60% confluence in 4-well glass chamber slides were incubated with DMEM containing CSFE-labeled EVs at a ratio 1 *μ*g EVs per 10000 adherent cells. At the end of incubation time (2, 4, and 8 hours), cells were washed twice with PBS and fixed with 4% paraformaldehyde/PBS for 10 minutes at room temperature. Nuclei were stained with DAPI (Sigma-Aldrich) and then mounted with Permafluor and a coverslip (Thermo Fisher Scientific). Cellular uptake of EVs was visualized under a Leica confocal station (Leica SP5 confocal system) mounted on a Leica DM6000 inverted microscope (Leica Microsystems Inc., Buffalo Grove, IL, USA).

### 2.11. Statistical Analysis

For NTA and miRNAs analysis, three fetal-derived and three adult-derived EV samples were analyzed. For *in vitro* angiogenesis, four different samples corresponding to treatment with fetal-derived EVs and three different samples corresponding to the remaining treatments (adult-derived EVs, fetal-derived CM, and the corresponding EV-depleted CM) were analyzed. For cell migration assay, three different samples of each condition were analyzed (fetal-derived EVs, adult-derived EVs, fetal CM, and the corresponding EV-depleted CM). For quantitative analysis by Luminex of whole CM vs. EV-depleted CM, ten fetal CM samples were analyzed. Data were analyzed with *R* [23] and expressed as the mean ± SD. Data from two different groups were compared with Student's *t*-test. Differences between the groups were considered significant at a *p* value of ≤ 0.05.

Angiogenesis data were analyzed with GraphPad Prism 8.4.2 and expressed as the mean ± SD. Data from each condition were compared to treatment with fetal-derived EVs with the one-way ANOVA.

## 3. Results

### 3.1. Characterization of EVs by Nanoparticle Tracking Analysis and Protein Marker Expression

**Figure 1:**
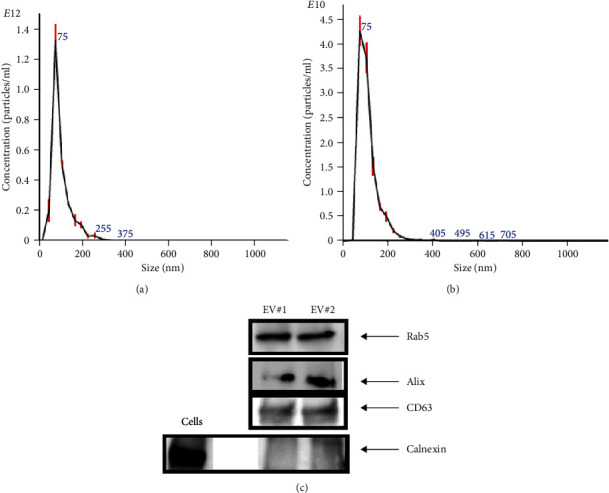
Physical characterization of EVs by NTA and protein expression. (a) Representative histogram of EVs isolated from secretome of fetal dermal cells showing a peak corresponding to a mode value of 77.5 ± 0.8 nm size and a concentration of 2.59 × 10^12^ particles/ml. (b) Representative histogram of EVs isolated from secretome of adult dermal cells showing a peak corresponding to a mode value of 87.2 ± 2.8 nm size and a concentration of 1.12 × 10^11^ particles/ml. The results shown are representative of three independent experiments. (c) Representative Western blot analysis of two fetal dermal-derived EV samples showing expression of EV markers Rab5, Alix, and CD63 in total protein extracts of pellet particles. Negative control, calnexin in cell protein extracts, and pellet particles are also shown. NTA: nanoparticle tracking analysis; EVs: extracellular vesicles; cells: total protein extracts of human fetal dermal cells; EV#1: sample 1; EV#2: sample 2.

The NTA of pellet preparations obtained by differential ultracentrifugation of CM from fetal and adult dermal cells indicated a “mode” diameter size (representing the size of the most abundant particles in a sample preparation) consistent with that of “small EVs” (<200 nm [24]). The mode sizes of a representative fetal sample (77.5 ± 0.8 nm) and that of a representative adult sample (87.2 ± 2.8 nm) are shown in Figure 1(a) and Figure 1(b), respectively. The concentration of EVs from both fetal and adult cells was in a range of 10^11^-10^12^ particles/ml. Moreover, characterization by Western blot analysis revealed that pellet particles expressed Rab5, Alix, and CD63 proteins, and while were negative for calnexin protein, this latter found in total protein extracts from human fetal dermal cells (Figure 1(c)).

### 3.2. Differential Expression of EV miRNAs and Their Association with Signaling Pathways Related to Angiogenesis and Wound Healing

We found 87 highly expressed miRNAs (Ct values ≤ 26) in fetal dermal cell-derived EVs. These highly expressed miRNAs were also considered significantly upregulated in fetal compared to adult dermal cell-derived EVs (Table 1; Supplemental Material S1). In addition, 21 miRNAs had a validated role in angiogenesis according to literature (Table 1; Supplemental Material S1).

**Figure 2:**
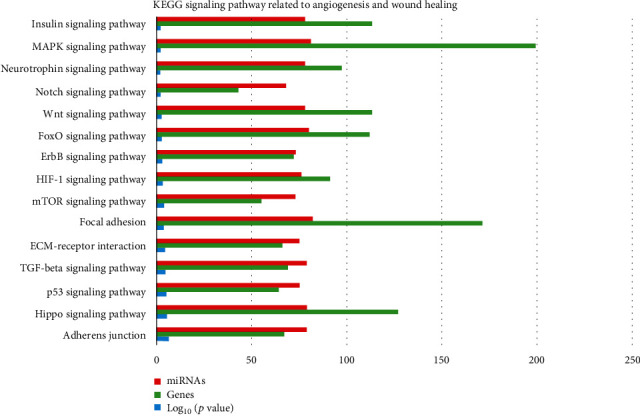
KEGG signaling pathways related to angiogenesis and wound healing obtained by screening out the 87 miRNAs considered significantly upregulated in fetal cell- vs. adult cell-derived EVs with the DIANA-miRPath v.3 software. The figure shows log_10_ (*p* value) (blue bars) associated with the number of miRNAs (red bars) targeting specific genes (green bars) within each pathway.

**Figure 3:**
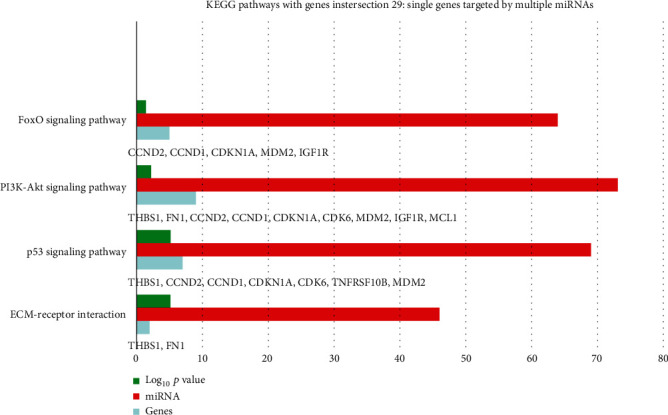
KEGG signaling pathways related to angiogenesis and wound healing obtained with genes intersection 29 to show putative genes targeted by multiple miRNAs within each pathway. DIANA tool was performed with the 87 miRNAs considered significantly upregulated in fetal dermal cell-derived EVs compared to adult dermal cell-derived EVs.

KEGG analysis with DIANA-TarBase of these 87 miRNAs evidenced 85 signaling pathways, 15 of which related to angiogenesis/wound healing (Table 2; Supplemental Material S2), (Figure 2). By setting 29 as a threshold (genes intersection) [20], we selected 4 signaling pathways associated with angiogenesis and wound healing from the obtained list (Figure 3; Supplemental Material S3). In particular, we found 46 out of 87 miRNAs enriched in ECM-receptor interaction signaling pathway, with two putative target genes, THBS1 and fibronectin (FN1) (Supplemental Material S3); 69 miRNAs enriched in the p53 signaling pathway with the target genes THBS1, cyclin D1 (CCND1), cyclin D2 (CCND2), cyclin-dependent kinase inhibitor 1 (CDKN1A), cell division protein kinase 6 (CDK6), TNF receptor superfamily member 10b (TNFRSF10B), and mouse double minute 2 homolog (MDM2) (S3); 73 miRNAs enriched in the PIK3/Akt signaling pathway with putative target genes THBS1, FN1, CCND2, CCND1, CDKN1A, CDK6, MDM2, insulin-like growth factor 1 receptor (IGF1R), and MCL1 (S3); 64 miRNAs enriched in the FoxO signaling pathway with the target genes CCND2, CCND1, MDM2, and IGF1R (S3).

### 3.3. Validation of the *In Silico* Analysis by Western Blot

**Figure 4:**
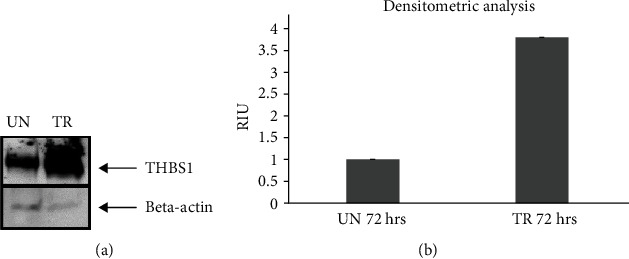
Validation of the *in silico* data. (a) Representative Western blot of THBS1 protein in total protein extracts of fibroblasts, untreated or treated with 10 *μ*g/ml of fetal-derived EVs for 72 hours. Beta-actin was used as internal loading control. (b) Densitometric analysis of the 72-hour time point. UN: untreated; TR: treated; THBS1: thrombospondin 1; EV: extracellular vesicle; RIU: relative intensity unit.

The amount of THBS1 protein in fibroblasts treated for 72 hours with 10 *μ*g/ml of fetal-derived EVs was higher than the amount of THBS1 protein in both untreated fibroblasts (Figure 4(a)) and the earlier time points of treatment (24 and 48 hours, data not shown). The amount of THBS1 protein in fibroblasts following a 72-hour treatment with fetal-derived EVs was approximately 4-fold higher than the amount of THBS1 protein in untreated fibroblasts at the same time point (Figure 4(b)).

### 3.4. EV/CM-Induced Mesh-Like Organization of HUVECs *In Vitro*

**Figure 5:**
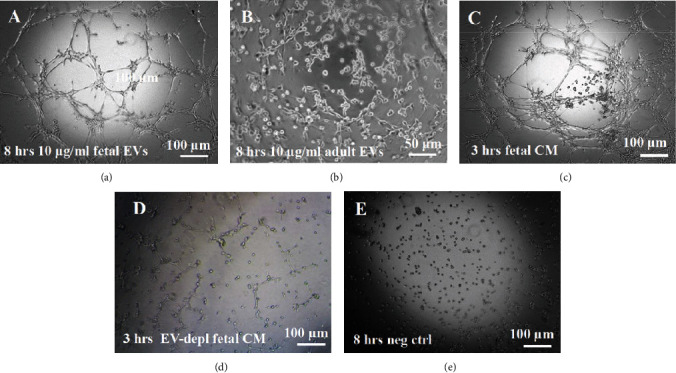
Effect of EVs on formation of mesh-like structures of HUVECs *in vitro*. (a) HUVECs in culture medium supplemented with 10 *μ*g/ml of fetal dermal cell-derived EVs 8 hours after plating on Matrigel. (b) HUVECs in culture medium supplemented with 10 *μ*g/ml of adult dermal cell-derived EVs 8 hours after plating. (c) HUVECs in fetal dermal cell-derived CM 3 hours after plating. (d) HUVECs in EV-depleted CM of fetal dermal cells 3 hours after plating. Negative control HUVECs in serum-free culture medium 8 hours after plating. Scale bars: 100 *μ*m (a, c, d, e) and 50 *μ*m (b). EV: extracellular vesicles; CM: conditioned medium; depl: depleted; neg. ctrl: negative control. The results shown are representative of four independent experiments.

**Figure 6:**
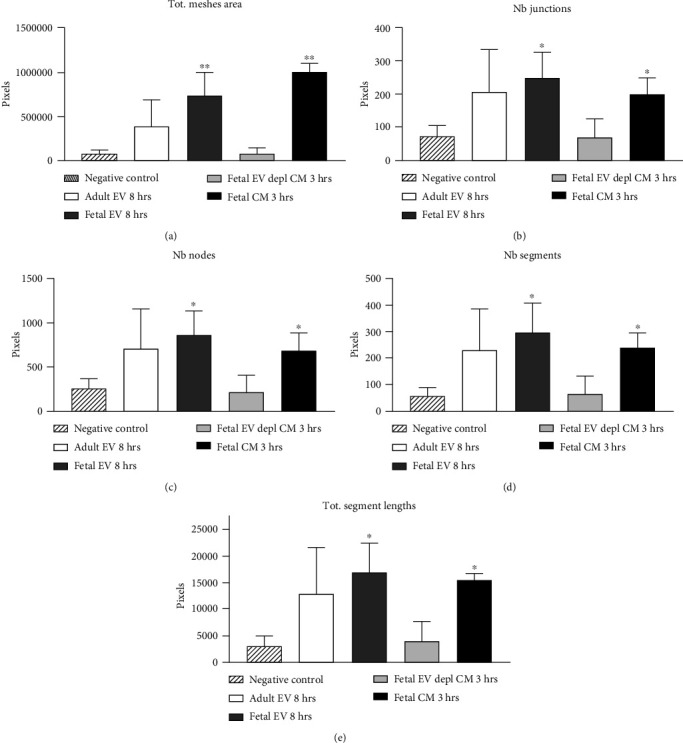
Angiogenic parameters quantified with the Angiogenesis Analyzer of ImageJ on images indicating (a) total mesh area, (b) number of junctions, (c) number of nodes, (d) number of segments, and (e) total segment length. 10 *μ*g/ml of EVs was always used. The images corresponded to the 3-hour time point for treatments with CM and to the 8-hour time point for treatment with EVs and for negative controls. Plotted values (mean ± SD) represent samples (*n* = 3 for each condition, except *n* = 4 for treatment with fetal dermal cell-derived EVs). ^∗^*p* ≤ 0.05; ^∗∗^*p* ≤ 0.001. Differences not denoted with an asterisk are not significant. Tot: total; EV: extracellular vesicle; depl: depleted; CM: conditioned medium; Nb: number.

HUVECs cultured in culture medium supplemented with 100, 50, or 10 *μ*g/ml of fetal dermal cell-derived EVs achieved the “complete mesh-like structures” pattern (maximum score 5) 8 hours after plating on Matrigel (Figure 5(a) obtained with 10 *μ*g/ml EVs), while only achieved the “sprouting of new capillary tubes” pattern (score 3) when cultured in the presence of 10 *μ*g/ml of adult dermal cell-derived EVs (Figure 5(b); Supplemental Material S4). The score 5 was achieved faster (approximately 3 hours) when cells were cultured in serum-free CM than in EVs of fetal dermal cells (Figure 5(c)). Interestingly, culturing HUVECs in EV-depleted CM only determined the achievement of score 3 (Figure 5(d)). Negative control HUVECs cultured in the absence of EVs maintained the “individual cells” pattern (score 0) for all the duration of the experiment (Figure 5(e)) (Table 3). Lower doses of fetal dermal cell-derived EVs (≤5 *μ*g/ml) were ineffective in inducing formation of mesh-like structures (data not shown). The results were confirmed by angiogenesis parameters quantified on images (*n* = 4 for treatment with fetal-derived EVs; *n* = 3 for the other treatments). In the case of HUVECs cultured in the presence of 10 *μ*g/ml of adult dermal cell-derived EVs, histograms were lower to those of HUVECs cultured in the presence of fetal cell-derived EVs, but differences were not statistically significant, probably due to the high SD values. Data were expressed as total mesh area (Figure 6(a)), number of junctions (Figure 6(b)), number of nodes (Figure 6(c)), number of segments (Figure 6(d)), and total length of segments (Figure 6(e)) (^∗^*p* value ≤ 0.05; ^∗∗^*p* value ≤ 0.001).

### 3.5. EV/CM-Induced Migration of Fibroblasts *In Vitro*

**Figure 7:**
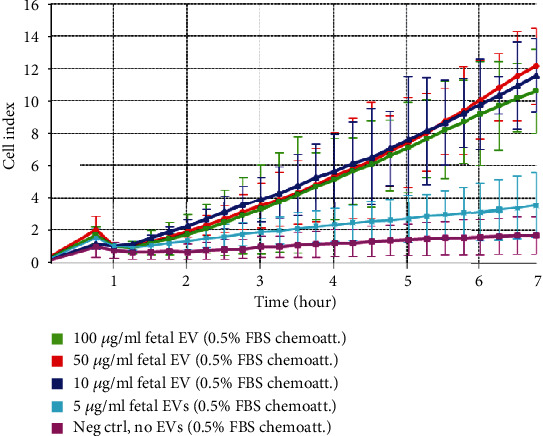
Effect of EVs on migration of fibroblasts *in vitro*. RTCA curves showing fibroblasts seeded in the presence of 100, 50, 10, and 5 *μ*g/ml of fetal dermal cell-derived EVs. Culture medium containing a small amount of FBS (0.5%) was used as chemoattractant. RTCA curve of negative control fibroblasts seeded without EVs is also shown. RTCA: real-time cell analyzer; EVs: extracellular vesicles; chemoatt: chemoattractant; FBS: fetal bovine serum; neg ctrl: negative control. The results shown are representative of six independent experiments.

Fibroblasts in the presence of 100, 50, or 10 *μ*g/ml of fetal dermal cell-derived EVs (green, red, and dark blue curves, respectively) migrated toward the lower chamber (containing culture medium 0.5% FBS as chemoattractant) with similar CI values. A lower concentration of EVs (5 *μ*g/ml) (light blue curve) resulted in the absence of migration, and CI values similar to those of negative control fibroblasts seeded in the absence of EVs (pink curve) (Figure 7). The CI values of cell migration induced by 10 *μ*g/ml of adult-derived EVs were similar to those induced by EVs of fetal origin (dark blue and orange curves, respectively) (data not shown).

### 3.6. Amount of Growth Factors and Chemokines in EV-Depleted CM

The levels of growth factors and chemokines in EV-depleted CM were similar to the levels in whole fetal dermal cell CM. The growth factor detected with the highest amount was VEGF-A, while the chemokine detected with the highest amount was SDF-1-alpha (Table 4).

### 3.7. Cellular Uptake of EVs

**Figure 8:**
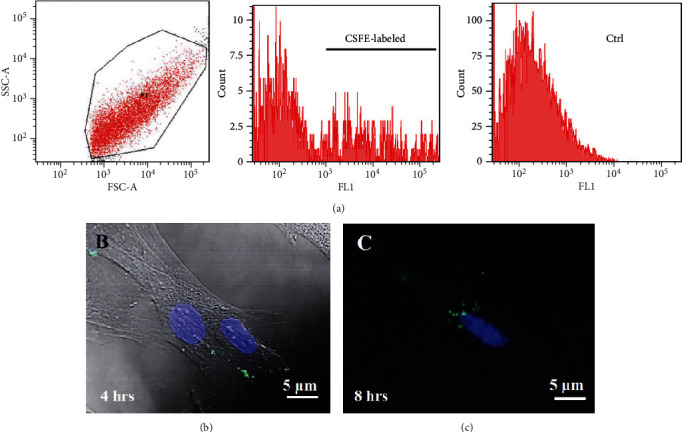
Internalization of CSFE-labeled EVs by targeted fibroblasts. (a) EVs analyzed by flow cytometry in a linear range for physical parameters FSC vs. SSC (forward scatter vs. side scatter) using Miltenyi beads as size marker. CSFE-labeled EVs and negative control, unlabeled EVs are also shown. Confocal images showing uptake of CSFE-labeled EVs by fibroblast target cells at 4- and 8-hour incubation times. (b) Bright field image merged with green (CSFE) and blue (DAPI) showing cytoplasmic localization of fluorescent signal at 4 hours of incubation. (c) Dual-channel confocal fluorescence showing cytoplasmic localization of fluorescent signal at 8 hours of incubation. The results shown are representative of three independent experiments. CSFE: carboxyfluorescein succinimidyl ester; EVs: extracellular vesicles; FL1: fluorescence 1. Scale bar: 5 *μ*m.

Labeling of fetal dermal cell-derived EVs with CSFE was successfully verified by flow cytometry. We clearly identified a discrete population of fluorescent particles in the range of small EVs compared to control, unlabeled EVs (Figure 8(a)). The fluorescent signal of CSFE-labeled EVs was visualized into the cytosol starting from 4 hours of incubation (Figure 8(b)), while at later time points (8 hours of incubation; Figure 8(c)), the signal was mainly detected in the perinuclear region of target cells. No differences were found when using targeted HUVECs or fibroblasts (data not shown).

## 4. Discussion

As a continuation of the previous study [12] and in search for the molecules contributing to functional activity of secretome, we herein focused on isolation and characterization of EVs from secretome of human fetal dermal cells. We used equal numbers of cultured cells and collected equal volumes of secretome at the same time, in the attempt to standardize the EV source. We followed the updated guidelines of the International Society for Extracellular Vesicles (ISEV), which recommend to use the generic term “EVs” for particles naturally secreted by cells, whose characterization is mainly based on physical parameters, such as size and concentrations [24]. The NTA of pellet particles obtained by ultracentrifugation of secretome of both fetal and adult dermal cells revealed a size diameter of approximately 70 nm, thus suggesting that we may have isolated “small EVs.” In fact, the general characterization suggested by the ISEV discriminates EV subtypes in small EVs (<200 nm size) and larger EVs [24]. Furthermore, the isolated EVs were positive for Rab5 and Alix, and CD63 while were negative for calnexin [24]. Since there is no a perfect quantification method, we followed the most common, which is based on total protein amounts in our dose-response studies [24].

Depletion of EVs by differential ultracentrifugation almost abrogated the *in vitro* proangiogenic effect of fetal dermal cell secretome. Since depletion of EVs only slightly reduced the efficiency of cell migration, we suggest that perhaps the amount of chemokines such as SDF-1 alpha and MCP-1 in EV-depleted secretome may be sufficient to ensure a migratory response. On the contrary, a VEGF-A concentration of approximately 5000 pg/ml in EV-depleted secretome could not be sufficient to ensure formation of mesh-like structures normally requiring higher doses of VEGF-A (e.g., 20000 pg/ml or more) [25]. Nevertheless, the multitude of factors contained in secretome is extremely difficult to establish which factor might be responsible for one activity or another.

According to the current version of the database Exocarta [26, 27], 9769 proteins, 1116 lipids, 3408 mRNAs, and 2838 miRNAs have been identified in EV/exosomes from several cell types and organisms. Research studies have often hypothesized that the transfer of miRNAs in particular will account for the understood EV-mediated effects [14]. miRNA-based therapy entered in clinical studies mainly for cancer treatments, while it is still in early stages for applications of regenerative medicine [28, 29]. However, emerging reports are available indicating the vast potential of miRNAs for the repair of several tissues including bone/cartilage muscle, cardiovascular tissue, neurological tissue, skin, and even in angiogenesis [29, 30]. With respect to organ repair and angiogenesis, the involvement of miRNAs in different phases of wound healing has been documented [31, 32], and several proangiogenic miRNAs have been identified and validated [33, 34] .

By analyzing the miRNA expression profile of dermal cell-derived EVs, we identified 87 miRNAs significantly upregulated in fetal- vs. adult-derived EVs, which included miRNAs validated in angiogenesis such as let-7b-5p, let-7g-5p, miRNA-10a, -15b, -16-5p, members of the cluster 17-92 (-17-5p, -19a-3p, -19b-3p, -20a-5p, -92a-3p), -21-5p, members of the cluster 23-27 (-24-3p, -27b-3p), -31-3p, -31-5p, -132-3p, -199a-3p, -218-5p, -221-3p, -222-3p, and -320a [29, 34–40] (Table 1). Since the depletion of EVs from fetal dermal cell secretome impaired the *in vitro* tube formation, we suggest that the proangiogenic effect of fetal dermal cell secretome reported in our previous study [12] could largely depend on an EV-mediated transfer of these miRNAs. Interestingly, significantly upregulated miRNAs in fetal- vs. adult cell-derived EVs also included proregenerative miRNAs such as -26a [41], -29b [42, 43], -132 [44], -193b-3p [45], and -199a-3p [46], which have been delivered in animal models to improve angiogenesis, bone and cartilage regeneration, cardiac regeneration, and for fibrosis treatment (Table 1).

According to KEGG analysis, 85 signaling pathways were detected as targets for the 87 miRNAs upregulated in fetal- vs. adult-derived EVs. Each of the pathways was targeted by multiple miRNAs, and 15 of the 85 pathways were associated with angiogenesis and wound healing. These 15 pathways included adherent junction [47], HIPPO [48–51], p53 [52], TGF-beta [53, 54], ECM-receptor interaction [55], focal adhesion [56], mTOR (interconnected to PI3K-Akt) [57–59], HIF-1 [60], ErbB [61], FoxO (upstream in the activation of TGF-beta and PI3k-Akt), Wnt [62], Notch [63–66], neurotrophin [67], MAPK [68], and insulin (upstream to mTOR) signaling pathways. Of particular interest was the HIPPO signaling, whose role in regulating regeneration of organs such as the intestine, liver, heart, nervous system, and skin is well documented. By setting genes intersection option (29), 10 putative target genes (THBS1, FN1, CCND1, CCND2, CDKN1A, CDK6, TNFRSF10B, MDM2, IGF1R, and MCL1) associated with 4 of the 15 signaling pathways (ECM-receptor interaction, PI3K/Akt, p53, and FoxO) were obtained and predicted as targets for our miRNAs. Among the putative target genes, we focused on THBS1 to validate the *in silico* analysis. THBS1 is a multifunctional extracellular matrix glycoprotein produced by several cell types, facilitating tissue repair in different healing models [69–71]. Western blot analysis showed a significant increase of THBS1 protein amounts (approximately 4-fold) in fibroblasts treated for 72 hours with fetal dermal-derived EVs against untreated fibroblasts.

Both freshly isolated and frozen EVs were tested in cell-based assays of angiogenesis and cell migration, thus showing no differences in their performances. This observation was in agreement with a previous report showing that the storage in the absence of cryoprotectant at -20°C did not affect the biochemical activity of EVs [72]. While EVs from both cell types stimulated cell migration with a similar efficacy, fetal dermal cell-derived EVs were far more effective than adult dermal cell-derived EVs in inducing formation of mesh-like structures. Even if this result was not statistically significant, the slide in Supplemental Material S4 clearly shows this difference. Overall, we observed a delay in EV-induced cellular responses compared to secretome/CM-induced cellular responses. Since the delay was independent from the used EV concentrations (e.g., either 100 or 10 *μ*g/ml), we suggest that the delay could be due to the dynamic of EV internalization by target cells.

In view of the promising prospective of miRNA-based therapeutics, the research is dedicated in solving some challenges in order to make it more translationally valuable. These challenges include the off-target effects of miRNA or their low internalization by target cells making their delivery difficult [73]. Typically, miRNAs for therapeutics have been delivered by direct injection, viral vectors, or coupled to scaffolds [74]. A number of studies indicate that EVs may exert their effect via horizontal transfer of their cargo [14]. Therefore, EVs could serve as a vehicle to successfully deliver miRNAs of interest to several therapeutic applications [75]. Confocal microscope observations of a punctuated green fluorescent pattern inside target cells suggest successful internalization of CSFE-labeled EVs. The signal was visualized in the cytosol of both HUVECs and fibroblasts at not earlier than 4 hours of incubation, and in the perinuclear region at later time points (from 8 hours of incubation). To conclude, the proangiogenic features of secretome of human fetal dermal cells appear largely related to the presence of small EVs. Although there is much to be learned in the field of EV research, the unique properties of these particles clearly represent a new therapeutic opportunity for tissue regeneration [15, 76], since it could offer a number of advantages over traditional cell transplantation as a cell-free product. As with conventional drugs, EVs can be standardized and tested in terms of dose and biological activity. Furthermore, EVs can be produced in clinical grade, freeze, and easily delivered. Finally, EV-based therapy could overcome the challenge of a successful delivery of miRNA molecules *in vivo* [77].

## Figures and Tables

**Table 1:** EV-derived miRNAs found highly expressed in fetal samples (Ct values ≤ 26), which were also upregulated compared to adult samples (see also Supplemental Material S1).

miRNA name	Role in angiogenesis/tissue regeneration	References	Fold increase in fetal vs. adult samples	*p* value
hsa-let-7b-5p	It targets VEGF gene; validated role in angiogenesis	Hua et al., 2006 [36]; Landskroner-Eiger et al., 2013 [34]	26.2091	0.0074
hsa-let-7g-5p	Validated role in angiogenesis	Landskroner-Eiger et al., 2013 [34]	175.3585	0.021
hsa-miR-10a-5p	Validated role in angiogenesis; angiogenesis influencer	Landskroner-Eiger et al., 2013 [34]	242.9505	0.03
hsa-miR-10b-3p			17.9198	0.0346
hsa-miR-15b-5p	Angiogenesis regulator; delivered in regenerative medicine for cardiac repair	Curtin et al., 2018 [29]; Wang & Olson, 2009 [39]	2624.268	0.0003
hsa-miR-16-5p	Angiogenesis regulator; it targets VEGF gene; regulator of angiogenesis; induces tube formation of HUVECs	Hua et al., 2006 [36]; Poliseno et al., 2006 [78]; Suarez & Sessa, 2009 [38]; Wang & Olson, 2009 [39]	149.6445	0.0183
hsa-miR-17-5p	Angiogenesis regulator; it targets VEGF gene	Caporali & Emanueli, 2011 [33]; Hua et al., 2006 [36], Wang & Olson, 2009 [39]	133.7475	0.0032
hsa-miR-19a-3p	Validated role in angiogenesis	Landskroner-Eiger et al., 2013 [34]; Wang & Olson, 2009 [39]	51.8807	0.0114
hsa-miR-19b-3p	Angiogenesis regulator	Caporali & Emanueli, 2011 [33]; Wang & Olson, 2009 [39]	109.3557	0.0044
hsa-miR-20a-5p	It targets VEGF gene; validated role in angiogenesis	Hua et al., 2006 [36]; Landskroner-Eiger et al., 2013 [34]; Wang & Olson, 2009 [39]	56.7629	0.0004
hsa-miR-21-5p	Angiogenesis regulator; wound healing regulator	Wang & Olson, 2009 [39]; Wang et al., 2012 [32]	157.6826	0.0046
hsa-miR-24-3p	Highly expressed by endothelial cells	Suarez & Sessa, 2009 [38]; Zhou et al., 2011 [40]	25.4984	0.0059
hsa-miR-26a-5p	Proregenerative (it promotes osteogenesis-angiogenesis in mouse)	Li et al., 2013 [47]	132.6638	0.0122
hsa-miR-26b-5p	Tissue repair; remodeling in wound healing	Banerjee & Sen, 2013 [31]; Sen et al., 2015 [30]	95.4933	0.0183
hsa-miR-27b-3p	It targets VEGF gene; validated role in angiogenesis	Hua et al., 2006 [36]; Landskroner-Eiger et al., 2013 [34]; Wang & Olson, 2009 [39]; Zhou et al., 2011 [40]	950.021	0.0096
hsa-miR-28-3p			49.9995	0.0082
hsa-miR-29b-3p	Regulator of tissue regeneration; proregenerative (delivered for ECM remodeling in fibrosis treatment)	Monaghan et al., 2014 [42]; van Rooij et al., 2008 [43]	61.9175	0.0105
hsa-miR-30a-3p	It targets VEGF gene; endothelial cell modulator	Bridge et al., 2012 [35]; Hua et al., 2006 [36]	185.51	0.0099
hsa-miR-30b-5p	Angiogenesis regulator; endothelial cell modulators; it targets VEGF gene	Bridge et al., 2012 [35]; Hua et al., 2006 [36]	32.3336	0.0004
hsa-miR-30c-5p	Endothelial cell modulator	Bridge et al., 2012 [35]	30.1373	0.0002
hsa-miR-30e-3p	Endothelial cell modulator	Bridge et al., 2012 [35]	41.2049	0.0041
hsa-miR-31-5p	Angiogenesis regulator; wound healing	Li et al., 2015 [79]; Wang et al., 2012 [32]	60.803	0.0027
hsa-miR-31-3p	Angiogenesis regulator; wound healing	Li et al., 2015 [79]; Wang et al., 2012 [32]	229.5474	0.0097
hsa-miR-34a-5p	It targets VEGF gene		2254.5936	0
hsa-miR-92a-3p	Validated role in angiogenesis	Curtin et al., 2018 [29]; Landskroner-Eiger et al., 2013 [34]; Wang & Olson, 2009 [39]	32.781	0.0001
hsa-miR-93-3p			149.3753	0.0065
hsa-miR-99b-3p	Angiogenesis promoter	Kane et al., 2012 [37]	37.2967	0.0134
hsa-miR-99b-5p	Angiogenesis promoter	Kane et al., 2012 [37]	41.1216	0.0031
hsa-miR-103a-3p			809.2304	0.008
hsa-miR-106a-5p	It targets VEGF gene; regulator of angiogenesis	Hua et al., 2006 [36]	67.8178	0.0047
hsa-miR-106b-5p	It targets VEGF gene; regulator of angiogenesis	Hua et al., 2006 [36]; Landskroner-Eiger et al., 2013 [34]	56.2099	0.0084
hsa-miR-125a-5p	Tube formation of HUVECs; it targets VEGF gene	Hua et al., 2006 [36]; Poliseno et al., 2006 [78]	128.8981	0.0061
hsa-miR-125b-5p	Angiogenesis regulator; tube formation of HUVECs	Poliseno et al., 2006 [78]; Zhou et al., 2015 [80]	18.6552	0.0041
hsa-miR-127-3p			19.2386	0.0001
hsa-miR-132-3p	Validated role in angiogenesis during chronic wound healing; proregenerative (it promotes angiogenesis in myocardial infarction)	Landskroner-Eiger et al., 2013 [34]; Li et al., 2017 [81]; Ma et al., 2018 [44]	3664.7066	0.0034
hsa-miR-136-3p			164.0836	0.0268
hsa-miR-138-5p			52.6792	0.0162
hsa-miR-145-5p	Angiogenesis regulator	Fan et al., 2012 [82]	42.2841	0.0067
hsa-miR-146a-3p	Wound healing (inflammatory phase)	Banerjee & Sen, 2013 [31]	172.0832	0.0135
hsa-miR-146b-3p	Wound healing (inflammatory phase); angiogenesis promoter	Ahn et al., 2013 [83]; Banerjee & Sen, 2013 [31]	203.771	0.0161
hsa-miR-149-5p	Scarless wound healing	Lang et al., 2017 [84]	37.5122	0.0102
hsa-miR-151a-5p			126.8167	0.005
hsa-miR-151a-3p			17.732	0.0246
hsa-miR-152-3p			20.0452	0.0097
hsa-miR-155-5p	Wound healing (inflammatory phase); amyotrophic lateral sclerosis; anti-inflammatory action; angiogenesis regulator	Banerjee & Sen, 2015 [31]; Curtin et al., 2018 [29]; Suarez & Sessa, 2009 [38]	32.5772	0.04
hsa-miR-181a-5p	Angiogenesis promoter	Kane et al., 2012 [37]	112.4827	0.0011
hsa-miR-186-5p			78.0074	0.0165
hsa-miR-191-5p	It may regulate the angiogenic actions of VEGF	Landskroner-Eiger et al., 2013 [34]	66.0569	0.0121
hsa-miR-193a-5p	It targets VEGF gene	Hua et al., 2006 [36]	108.2673	0.0061
hsa-miR-193b-3p	Proregenerative (chondrogenesis)	Meng et al., 2018 [45]	16.6788	0.0057
hsa-miR-197-3p			47.9232	0.01
hsa-miR-199a-3p	It targets VEGF gene; proregenerative (cardiac regeneration)	Hua et al., 2006 [36]; Lesizza et al., 2017 [46]	79.9505	0.0112
hsa-miR-214-3p			34.8695	0.0131
hsa-miR-214-5p	It targets VEGF gene	Hua et al., 2006 [36]	493.6614	0.0151
hsa-miR-218-5p	Validated role in angiogenesis	Landskroner-Eiger et al., 2013 [34]	32.9227	0.0092
hsa-miR-221-3p	Validated role in angiogenesis	Landskroner-Eiger et al., 2013 [34]	27.5391	0.0003
hsa-miR-222-3p	Angiogenesis in wound healing; validated role in angiogenesis	Banerjee & Sen, 2015 [31]; Landskroner-Eiger et al., 2013 [34]	38.7974	0.0103
hsa-miR-224-5p			21.5539	0.0039
hsa-miR-320a	It targets VEGF gene; validated role in angiogenesis	Hua et al., 2006 [36]; Landskroner-Eiger et al., 2013 [34]	56.2268	0.0086
hsa-miR-323a-3p			686.8069	0.0163
hsa-miR-324-3p			137.7004	0.0144
hsa-miR-331-3p	It targets VEGF gene	Hua et al., 2006 [36]	43.6136	0.0043
hsa-miR-342-3p			169.8914	0.0114
hsa-miR-345-5p			200.3964	0.0134
hsa-miR-365a-3p			526.7213	0.0115
hsa-miR-370-3p			99.3169	0.0081
hsa-miR-374a-5p			44.7501	0.0225
hsa-miR-376a-3p			216.5826	0.0246
hsa-miR-376c-3p			216.5826	0.0246
hsa-miR-382-5p			6102.4887	0.0009
hsa-miR-409-3p			173.9373	0.0097
hsa-miR-411-5p			99.6678	0.0353
hsa-miR-424-3p			482.6326	0.0137
hsa-miR-432-5p			125.5471	0.0385
hsa-miR-433-3p			778.7489	0.0057
hsa-miR-455-5p			611.2326	0.0122
hsa-miR-484			85.625	0.0066
hsa-miR-487b-3p			131.5413	0.0028
hsa-miR-493-3p			1303.6278	0.0053
hsa-miR-532-3p			42.572	0.0015
hsa-miR-532-5p	Angiogenesis	Slater et al., 2018 [85]	1363.2918	0.0072
hsa-miR-539-5p			350.9595	0.0063
hsa-miR-574-3p			59.5134	0.0121
hsa-miR-625-3p			77.7476	0.002
hsa-miR-708-5p			59.2604	0.0013
hsa-miR-766-3p			1533.4818	0.016
hsa-miR-886-5p			75.0277	0.0048
hsa-miR-1290			24.3418	0.0021

Plotted values (mean ± SD) represent fetal samples (*n* = 3) compared to adult samples (*n* = 3).

**Table 2:** KEGG signaling pathways and their association with angiogenesis and wound healing.

KEGG signaling pathway	Role in angiogenesis/wound healing	References	log_10_ (*p* value)
Adherens junction	Wound closure	Fenteany et al., 2000 [47]	5.10*E* − 07
HIPPO	Organ regeneration	Juan & Hong, 2016 [48]; Lee et al., 2014 [49]; Zhao et al., 2011 [51]; Wang et al., 2017 [50]	3.73*E* − 06
p53	Promotes VEGF expression and angiogenesis	Farhang Ghahremani et al., 2013 [52]	9.63*E* − 06
TGF-beta	Skin wound healing	Finnson et al., 2013 [53]; Ramirez et al., 2014 [54]	2.39*E* − 05
ECM-receptor interaction	Wound repair	Olczyk et al., 2014 [55]	4.53*E* − 05
Focal adhesion	Cell migration; angiogenesis	Zhao & Guan, 2011 [56]	0.000175201
mTOR	Interconnected to PI3K-Akt pathway to accelerate epithelial wound healing; angiogenesis	Castilho et al., 2013 [56]; Karar & Maity, 2011 [59]	0.000176029
HIF-1	Accelerating wound healing by enhancing angiogenesis	Hong et al., 2014 [60]	0.000538735
ErbB	Mediates proliferation and migration of keratinocytes in wound healing (ErbB1)	Pastore et al., 2008 [61]	0.001490837
FoxO	Upstream in the activation of both TGF-beta and PI3K-Akt signaling pathways		0.002880647
Wnt	Participates to each stage of the healing process	Whyte et al., 2012 [62]	0.00326995
Notch	Angiogenesis and endothelial cell formation; essential in organ regeneration; vasculature repair after brain trauma and wound healing	Carlson et al., 2007 [63]; Raya et al., 2003 [66]; Ran et al., 2015 [65]; Chigurupati et al., 2007 [64]	0.004876559
Neurotrophin	Novel regulator of angiogenesis	Kraemer & Hempstead, 2003 [67]	0.009582793
MAPK	Skin reepithelialization	Deng et al., 2006 [68]	0.008652606
Insulin	Upstream to PI3k/Akt and mTOR	Karar & Maity, 2011 [59]	0.014139516

DIANA tool analysis of the 87 miRNAs considered significantly upregulated in fetal vs. adult dermal cell-derived EVs.

**Table 3:** Numerical value assigned to each pattern associated with the degree of *in vitro* angiogenesis. A representative sample for each condition is shown, corresponding to Figure 5.

Sample	Pattern	Score
Fetal EVs	Complete mesh-like structures	5
Adult EVs	Sprouting of new capillary tubes	3
Fetal CM	Complete mesh-like structures	5
EV-depleted fetal CM	Sprouting of new capillary tubes	3
Negative control	Individual cells, well separated	0

EV: extracellular vesicles; CM: conditioned medium.

**Table 4:** Customized ProcartaPlex human growth factor and chemokine panel. Amount of soluble factors in EV-depleted vs. whole CM of fetal dermal cells.

Soluble factor	Role in MSC-mediated wound healing	EV-depleted CM (pg/ml/10^6^ cells/24 h)	Whole CM (pg/ml/10^6^ cells/24 h)
VEGF-A	Angiogenesis [86]	6049 ± 1603	5899 ± 618
HGF	Epithelialization, neovascularization [87]	1031 ± 246	1448 ± 121
SDF-1 alpha (CXCL-12)	Angiogenesis [88]; cell migration [89]	6016 ± 1860	5983 ± 231
MCP-1 (CCL-2)	Angiogenesis [1]; recruitment of neutrophils [90]; remodeling [91]	1771 ± 795	1233 ± 54
IL-8	Recruitment of neutrophils, epidermal cell migration, angiogenesis [91]	1546 ± 293	788 ± 697
GRO-alpha (CXCL-1)	Recruitment of neutrophils [91]; angiogenesis [1]	907 ± 328	973 ± 100

Plotted values (mean ± SD) represent EV-depleted CM (*n* = 10) compared to whole CM (*n* = 10). Differences not denoted with an asterisk are not significant. EV: extracellular vesicle; CM: conditioned medium.

## Data Availability

The data concerning the NTA of EVs, the heat map graph, the figures and graphs of in vitro angiogenesis, the curves of cell migration, the flow cytometry graphs of CSFE-labeled EVs, and the uptake of EVs used to support the findings of this study are included within the article. The miRNA data used to support the findings of this study are included as a table within the articles, but also in supplementary information file S1 The KEGG data used to support the findings of this study are included within the article (as graphs), and also in supplementary information files S2 and S3.

## Supplementary Materials

Supplementary Materials S1 (excel file): list of significantly upregulated miRNAs in fetal vs. adult EVs; a list of all analyzed miRNAs is also included. S2 (excel file): complete list of the KEGG signaling pathways; list of the KEGG signaling pathways associated with angiogenesis and wound healing. S3 (excel file): complete list of the KEGG signaling pathways obtained with genes intersection 29, including putative target genes. S4 (power point slide): pictures of HUVECs treated with fetal-derived EVs (*n* = 4) and adult-derived EVs (*n* = 3). Each picture represents an independent experiment.
